# Predicting vertical ground reaction force characteristics during running with machine learning

**DOI:** 10.3389/fbioe.2024.1440033

**Published:** 2024-10-08

**Authors:** Sieglinde Bogaert, Jesse Davis, Benedicte Vanwanseele

**Affiliations:** ^1^ Human Movements Biomechanics Research Group, Department of Movement Sciences, KU Leuven, Leuven, Belgium; ^2^ Department of Computer Science, Leuven.AI, KU Leuven, Leuven, Belgium

**Keywords:** running, machine learning, vertical ground reaction force, inertial measurement unit, contact time, active peak, impact peak, impulse

## Abstract

Running poses a high risk of developing running-related injuries (RRIs). The majority of RRIs are the result of an imbalance between cumulative musculoskeletal load and load capacity. A general estimate of whole-body biomechanical load can be inferred from ground reaction forces (GRFs). Unfortunately, GRFs typically can only be measured in a controlled environment, which hinders its wider applicability. The advent of portable sensors has enabled training machine-learned models that are able to monitor GRF characteristics associated with RRIs in a broader range of contexts. Our study presents and evaluates a machine-learning method to predict the contact time, active peak, impact peak, and impulse of the vertical GRF during running from three-dimensional sacral acceleration. The developed models for predicting active peak, impact peak, impulse, and contact time demonstrated a root-mean-squared error of 0.080 body weight (BW), 0.198 BW, 0.0073 BW ⋅ seconds, and 0.0101 seconds, respectively. Our proposed method outperformed a mean-prediction baseline and two established methods from the literature. The results indicate the potential utility of this approach as a valuable tool for monitoring selected factors related to running-related injuries.

## 1 Introduction

Recreational distance running is a popular form of physical activity that offers numerous health benefits ranging from improvements in mental health (Oswald et al., 2020) to a lower risk of all-cause or cardiovascular mortality (Lee et al., 2017; Schnohr et al., 2013; Lavie et al., 2015). However, running also creates the risk of developing running-related injuries (RRIs) such as medial tibial stress syndrome, Iliotibial band syndrome, and Achilles tendinopathy, among others (Lopes et al., 2012). The incidence of RRIs in novice and recreational runners are 17.8 and 7.7 per 1,000 h of running respectively (Videbæk et al., 2015). About 75%–80% of RRIs are overuse injuries (Hespanhol Junior et al., 2017; Walther et al., 2005), which are a consequence of an imbalance between the cumulative musculoskeletal loading load and the runners’ load capacity (Bertelsen et al., 2017). Therefore, monitoring the musculoskeletal load during running is essential to prevent RRIs (Johnson et al., 2020).

A surrogate for the musculoskeletal load is the whole-body biomechanical load, which can be extrapolated from ground reaction forces (GRFs). The vertical GRF (vGRF) force-time curve exhibits characteristics linked to the risk of running-related injuries, which we will examine. In rearfoot-strike runners, the vertical GRF (vGRF) force-time curve displays an impact peak within the first 10% of the stance phase, followed by a larger active peak around halfway through the stance phase. Higher active peaks have been associated with an increased risk of RRIs, for example, a stress fracture (Popp et al., 2017; Grimston et al., 1991; Kliethermes et al., 2021), patellofemoral pain (Messier et al., 1991), and ankle instability (Bigouette et al., 2016). While some studies (Messier et al., 2018; Bredeweg et al., 2013; Davis et al., 2016) observed no differences in the impact peak between injured and non-injured runners, undergoing a gait-retraining program targeting the reduction of the impact peak (Chan et al., 2018) has shown to reduce a runner’s chance of sustaining an injury. The differences in ground contact time among injured and non-injured male novice runners (Bredeweg et al., 2013) and the association between vGRF impulse and bone stress injuries (Kliethermes et al., 2021), further indicate the importance of vGRF characteristics in assessing the risk of RRIs. Despite these results, inconsistencies persist in the relationship between vGRF characteristics and RRIs. One contributing factor may be that these studies only capture a snapshot of the load during running in a controlled environment. A more comprehensive understanding of the relationship between load throughout the training program and the development of RRIs requires incorporating load measures in real-life settings.

The conventional method for measuring GRFs during running relies on an expensive instrumented treadmill installed in a laboratory setting. While this method is accurate (Kram et al., 1998), it is impractical for real-world environments. In contrast, inertial measurement units (IMUs) consisting of an accelerometer, a gyroscope, and a magnetometer, facilitate monitoring runners in their natural environment. IMUs have already shown their potential in estimating runners’ fatigue status (Op De Beéck et al., 2018), gait kinematics (Wouda et al., 2018), and injury status (Bogaert et al., 2022). IMUs can also be used to indirectly estimate GRFs with studies proposing approaches for the impact peak, active peak, loading rate, and impulse of the vGRF (Verheul et al., 2019; Alcantara et al., 2021; Wouda et al., 2018; Seeley et al., 2020). One approach estimates these characteristics using an alternative expression of Newton’s second law, where the vGRF is approximated from the sum of the product of the masses and the acceleration of body segments (Verheul et al., 2019). Including more segments increases this approach’s accuracy but comes with added financial expenditures, logistical complexities, and discomfort. An alternative to the physics-based approach in a single-IMU setting is to use machine learning to predict the characteristics of the vGRF (Alcantara et al., 2021; Wouda et al., 2018; Donahue and Hahn, 2023).

While the machine-learning approaches are appealing, they still have several shortcomings. First, while they may be more accurate than the physics-based approach in a single-IMU setting, they do not reach the level of performance required for all applications. For example, detecting changes in vGRF active and impact peaks attributed to chronic ankle instability requires an accuracy of 0.095 body weight (BW) and 0.18 BW respectively, which these models do not achieve (Bigouette et al., 2016). Second, they focus on domain-specific (e.g., acceleration-based-estimated active peak, step frequency, and speed) and subject-specific (e.g., body mass) features, ignoring automatically-extracted features, which have proven to improve performance (Bogaert et al., 2022). Third, previous studies focused on a narrowly defined population of elite athletes (Alcantara et al., 2021; Verheul et al., 2019; Komaris et al., 2019). Hence, it is unclear if these approaches are applicable to the broader population. Finally, previous studies have not compared the developed approaches on the same dataset. This raises concerns about the reliability of the comparisons because the considered dataset can have a large effect on the findings.

This study aims to develop a new machine-learning approach that results in more accurate predictions of vGRF characteristics—active peak, impact peak, impulse of stance phase, and contact time—during running and tackles the abovementioned shortcomings. In addition, we will compare this method with existing methods on the same dataset.

## 2 Methods

### 2.1 Participants

Forty-three subjects, 31 males and 12 females, with varying running experience and sports engagement participated in the study. The social and societal ethics committee at the KU Leuven approved this study (G-2022-5367-R4) and every participant signed an informed consent.

### 2.2 Data collection

Every participant walked and ran on a treadmill for 11 min to warm up and familiarize with running on a treadmill. Afterward, the subject ran in random order at 2.22 m/s, 2.50 m/s, 2.78 m/s, 3.33 m/s, self-reported preferred speed for a 5,000 m run, preferred speed–0.14 m/s, and preferred speed +0.14 m/s. The subjects skipped any speeds they were uncomfortable with and did not repeat any speeds.

The Xsens link system (Xsens Technologies, Movella; Enschede, the Netherlands) collected 3D acceleration at 240 Hz and was secured using straps, a closely-fitted T-shirt, and a belt over the L3 to L5 spinal segments for the pelvis sensor. An instrumented treadmill (Motek, Motek Medical B.V.; Houten, the Netherlands) captured ground reaction forces at a sampling rate of 1,200 Hz (or 1,000 Hz for one subject). We synchronized the vertical ground reaction force and acceleration data using a marker positioned on or next to the pelvis sensor monitored by a 3D motion capture system (Vicon Motion Systems; Los Angeles, United States) at a sampling rate of 120 Hz (or 100 Hz for one subject).

### 2.3 Data preprocessing

Following the exclusion of data-corrupted trials, the dataset comprises 234 trials across 43 subjects. The ground reaction force of the remaining trials was filtered using a fifth-order Butterworth low-pass filter with a 30 Hz cutoff frequency and normalized to the participant’s body weight. We identified steps by localizing where the vGRF intersects the 50 N threshold (Vanwanseele et al., 2020). For each detected step, we calculated the ground truth value of the active peak, impact peak, impulse of the stance phase, and contact time. The acceleration signals were filtered with a fifth-order Butterworth low-pass filter with a cutoff value of 60 Hz. The detection of initial contact and the toe-off event was based on the acceleration signal crossing the threshold of 0.18 g (with g = 9.81 m/s^2^) and −0.25 g along with a set of constraints concerning the expected increase after initial contact and decrease before initial contact and toe-off event. When no toe-off event was found for a detected step, the threshold of −0.25 g was increased. Acceleration-detected steps that could not be matched to a corresponding GRF-detected step, steps with a stance phase longer than 0.4 s or shorter than 0.167 s, or for which there was suspicion of data corruption (e.g., signal falling away) were omitted. Overall, we obtained 92,974 steps of which 38,245 had an impact peak.

### 2.4 Feature extraction and model

For each step, we calculated a set of features, which can be divided into three categories:

**Subject-specific features** include mass and leg length (based on the distance from the hip to the ground during standing).

**General time-series features** are constructed using the TSFresh package (Christ et al., 2018). The Supplementary Material shows the considered features.

**Domain-specific time-series features** include step frequency, impulse of vertical acceleration data during the stance phase, and impulse of the entire step of the vertical acceleration data.

We eliminated features with a missing value or that always take on the same value. Categorical features are one-hot encoded and numerical features are standardized within a cross-validation approach. Next, we used a Lasso (Least Absolute Shrinkage and Selection Operator) model to predict the vGRF characteristics with a regularization strength between $5\cdot 10^{-6}$ and 0.05.

Including running speed as an input feature allows the model to account for the variations tied to changes in speed, particularly if running speed is relevant to the prediction task. However, to also account for the limited availability of accurate running speed outside a controlled environment, we trained the models with and without speed as an input parameter.

### 2.5 Method comparison

We compared our proposed approach to Verheul et al. (2019)’s method for predicting the impact peak, and Alcantara et al. (2021)’s approach for predicting the active peak, impulse, and contact time. Finally, we considered the mean regressor, a model that always predicts the mean value of the target variable as computed on the training data.

In Alcantara et al. (2021)’s method, active peak, contact time, impulse, and step frequency are derived from the estimated vGRF, calculated by the product of sacral acceleration and body mass. These acceleration-derived characteristics serve, along with body mass and speed, as input for a linear regression model or a quantile regression forest. Given the original paper’s results favoring linear regression over a quantile regression forest, we chose the linear regression model for implementing the Alcantara et al. (2021)’s method. Alcantara et al. (2021)’s method used, just like our method, 3D acceleration data collected from a sacral IMU.

In Verheul et al. (2019)’s method, a linear regression model utilizes the impact peak of the curve derived from the sum of the product of segments’ mass and acceleration to estimate the impact peak of the vGRF. To ensure a fair comparison with our method, we used for Verheul et al. (2019)’s method the 3D acceleration from a single position. Specifically, we selected the 3D trunk center of mass (CoM) acceleration data, the optimal segment for their single-segment model. The trunk’s CoM acceleration was approximated by linearly interpolating between the acceleration at the base of the neck and the base of the L5 disc which could, in principle, be captured by a single accelerometer. We used anatomical data to determine the position of the mass and CoM of the trunk (Plagenhoef et al., 1983). Verheul et al. (2019) used marker trajectories to derive the acceleration of the CoM of different segments. However, we adapted this to using acceleration measured by IMUs.

### 2.6 Model training and evaluation

We partitioned the data into a train and test set on subject level. There were 36 subjects in the train set and seven subjects in the test set. To select the hyperparameter settings and the set of features, we conducted a leave-one-subject-out cross-validation on the train set. Finally, the trained model was evaluated on the test dataset. All models were assessed using the root-mean-square error (RMSE) to quantify the difference between the predicted and observed values. In addition, we reported the mean-absolute-percentage error (MAPE) and the determination coefficient $\left(R^{2}\right)$ . This model training and evaluation process is consistently applied across all methods.

We executed all data processing, model training, and model evaluation in Python 3.10.9, except for the Alcantara et al. (2021)’s model, which was trained and evaluated using R 4.2.2.

## 3 Results

### 3.1 Participants

Table 1 summarizes the descriptive characteristics of the 43 participants. The participants’ ages ranged between 19 and 58 years, and their sports participation level varied from no sport to running more than 100 km a week.

**TABLE 1:** Descriptive characteristics of all participants included in the analysis. Values of continuous variables are expressed as mean ± standard deviation.

	All	Male	Female
Number of participants	43	31	12
Age (years)	24.7 ± 7.8	24.3 ± 6.6	25.8 ± 10.4
Mass (kg)	74.0 ± 11.9	79.2 ± 9.4	60.7 ± 5.5
Length (cm)	181.1 ± 10.2	185.6 ± 7.6	169.3 ± 5.9

### 3.2 Model performance

Across all steps of all participants, the mean ± standard deviation of the active peak, impact peak, and vGRF impulse, and contact time is 2.44 ± 0.24 body weight (BW), 1.65 ± 0.26 BW, 0.37 BW ⋅ s ± 0.019 BW ⋅ s, and 0.26 s ± 0.03 s, respectively.

Table 2 shows the performance of the vGRF characteristics prediction models. For each model, the validation score, used for hyperparameter tuning, and test score, used for model evaluation, are reported. For each characteristic, four different models are compared: (1) and (2) our method with and without speed as an input parameter, (3) the comparison method, and (4) a mean regressor. Our impulse-prediction model uses automatic-extracted features from the entire step, while our other models employ automatic-extracted features from the stance phase–a decision guided by validation scores.

**TABLE 2:** Root-mean-square error (RMSE), mean-absolute-percentage error (MAPE) and determination coefficient $\left(R^{2}\right)$ of our method (with and without speed as input), a comparison method, and a mean regressor to predict different characteristics of vGRF. The results of the validation set are a mean of the validation score over all folds of the cross-validation procedure.

Characteristic (unit)	Data set	Our method no speed			Our method with speed			Comparison method			Mean regressor		
		RMSE	MAPE	*R* ^2^	RMSE	MAPE	*R* ^2^	RMSE	MAPE	*R* ^2^	RMSE	MAPE	*R* ^2^
Active peak (BW)	Validation	0.106	3.59		0.099	3.33		0.173	6.31		0.225	8.14	
	Test	0.080	2.42	0.83	0.083	2.57	0.82	0.157	5.48	0.44	0.216	7.03	−0.22
Impact peak (BW)	Validation	0.172	9.22		0.168	9.01		0.255	13.88		0.259	14.24	
	Test	0.198	8.29	0.16	0.204	8.71	0.11	0.275	11.88	−0.62	0.266	11.53	−0.51
Impulse (BW ⋅ s)	Validation	0.0075	1.59		0.0075	1.59		0.0109	2.37		0.0181	4.35	
	Test	0.0073	1.53	0.79	0.0075	1.55	0.78	0.0087	1.78	0.71	0.0160	3.61	−0.01
Contact time (s)	Validation	0.0126	3.93		0.0109	3.45		0.0146	4.80		0.0301	10.07	
	Test	0.0101	3.01	0.89	0.0106	3.29	0.87	0.0125	3.89	0.83	0.0309	10.63	−0.07

Overall, the results of Table 2 indicate that our models performed better than the corresponding comparison method from the literature and the baseline (i.e., the mean regressor). Including speed in the feature set of our model did not improve the performance of the models.

Table 3 presents the performance of our model (without speed as an input), trained on data of different running speeds, but evaluated on subsets of the test set according to speed. The performance of the models, evaluated by RMSE, generally declines as speed increases, except for the contact-time model. A minor dip in RMSE is observed at 2.50 m/s for the active peak.

**TABLE 3:** Performance of our model (without speed as input) on speed-grouped subsets of the test set. Abbreviations: Root-mean-square error (RMSE) and mean-absolute-percentage error (MAPE).

Speed (m/s)	Active peak		Impact peak		Impulse		Contact time	
	RMSE	MAPE	RMSE	MAPE	RMSE	MAPE	RMSE	MAPE
2.22	0.0704	2.36	0.164	8.62	0.00635	1.32	0.0128	3.27
2.50	0.0653	2.13	0.172	8.96	0.00661	1.35	0.0084	2.22
2.78	0.0715	2.28	0.182	8.15	0.00746	1.56	0.0097	3.00
3.33	0.0948	2.86	0.279	10.5	0.00876	1.92	0.0118	4.08

## 4 Discussion

We developed models to predict the active peak, impact peak, impulse, and contact time during running from 3D acceleration collected by a sacral-mounted IMU. Our models performed better than the corresponding comparison method from the literature and the baseline (i.e., the mean regressor).

Our no-speed model for predicting the active peak of the vGRF obtained an RMSE of 0.080 BW and a MAPE of 2.42%, indicating that, on average, the model’s predictions were 2.42% off from the actual active-peak values. The RMSE score corresponds to an improvement of 0.077 BW compared to the Alcantara et al. (2021)’s model and 0.136 BW with respect to the mean regressor. The improvement in the coefficient of determination further underscores the enhanced performance of our model compared to the Alcantara et al. (2021)’s and baseline model. Similarly, our model’s contact time prediction accuracy, measured by RMSE, was 0.0024 s better than the Alcantara et al. (2021)’s method. For the impulse, our and Alcantara et al. (2021)’s model can accurately predict the impulse, yet our model has reduced the RMSE by 0.0014 BW ⋅ s and improved the $R^{2}$ by 0.08. Overall, we see a clear improvement in error compared to the Alcantara et al. (2021)’s model. One possible reason for the better results with respect to the Alcantara method is that the automatically-extracted features implemented in our model provide useful information.

Regarding contact time, Alcantara et al. (2021)’s method applied to our dataset yielded RMSE, MAPE, and $R^{2}$ values consistent with those reported in the original paper, indicating the generalizability of these models. We obtained slightly worse metric values for Alcantara et al. (2021)’s method for the active peak compared to those reported in the original paper. Our evaluation of the impulse model yielded an RMSE and MAPE over three times larger than the originally reported results. This inconsistency may be attributed to various factors, including the diverse characteristics of our participants, sample size, and running speeds. In light of these findings, it is crucial for forthcoming studies to examine the method’s generalizability and robustness.

We compare our model for estimating the impact peak with the single-IMU version of Verheul et al. (2019)’s method. As the reasoning behind the Verheul et al. (2019)’s model is based on the use of acceleration data of multiple segments, it has a relatively high RMSE for a single-IMU setting. Our model outperforms Verheul et al. (2019)’s approach and the mean regressor yielding an improvement in the RMSE of 0.077 BW and 0.068 BW respectively. The low determination coefficient for the impact-peak models and the high MAPE indicate the low quality of the prediction. This highlights that more research is needed to find a more suitable set of features and models for predicting the impact peak of the vGRF.

Since our models and the comparison models are trained and evaluated on the same dataset, differences in performance are not attributed to the dataset itself but rather to factors such as feature selection and model architecture. For all four vGRF characteristics, the superior performance of our method over the baseline model underscores the informativeness of the features in capturing deviations from the average.

Literature indicates that, in general, as running speed increases, contact time decreases. Concurrently, there is an increase in active peak, impact peak, and impulse (Nilsson and Thorstensson, 1989). Consequently, we expected that speed would be a valuable feature for predicting all characteristics. However, including speed in the feature set of our model to predict these characteristics didn’t have a large influence. A possible explanation for why including speed did not improve the predictions for the active peak, impact peak, impulse, and contact time models is that speed is potentially correlated with another considered feature based on the accelerometer signal. In addition, adjustments in running style in response to increased speed can vary on an individual level. Some runners might increase their stride length, others their stride frequency, which also has variable effects on the vGRF, complicating the relationship between speed and vGRF. Considering these outcomes alongside the general lack of readily available accurate speed information, incorporating speed into the model does not seem to be crucial for predicting vGRF characteristics.

The model’s evaluation on speed-grouped subsets of the test set (see Table 3) shows that the performance of the model for active peak, impact peak, and impulse decreases slightly with increasing speed. Apart from minor fluctuations, similar trends were seen for active peak (Donahue and Hahn, 2023; Patoz et al., 2022; Komaris et al., 2019) and impulse (Donahue and Hahn, 2023) in earlier studies. For the contact time, the model performs best at the intermediate speed, similar to the trend observed in a study by Patoz et al. (2022). Despite the observed trends, the variations are minimal and our models perform robustly across the speeds (except the impact-peak model for 3.33 m/s). This highlights the potential usefulness in a variety of practical applications.

The suitability of a model for a given application depends on achieving the required accuracy of that application. We evaluated our active- and impact-peak models for the ability to detect changes in active peak and impact peak due to chronic ankle instability, aiming for an RMSE of 0.095 BW for active peak and 0.18 BW for impact peak, based on half of the reported differences of 0.19 BW and 0.36 BW respectively (Bigouette et al., 2016). Our method meets the accuracy requirement for predicting the active peak, in contrast to the comparison method. However, for the impact peak, neither our nor the comparison method predicts the impact peak short with adequate accuracy, indicating the need for further improvement.

Regarding the impulse models, our and Alcantara et al. (2021)’s models are accurate enough to observe half of the difference in impulse after a 6-week forefoot strike intervention in runners with exertional compartment syndrome (Diebal et al., 2012).

For contact time, the practical threshold is 0.01 s, based on half the difference in contact time between the 40th and eighth kilometer of a marathon (Chan-Roper et al., 2012). Our no-speed model has a performance close enough (RMSE of 0.0101 s) to be considered acceptable for this practical application. In the Supplementary Material, the results and thresholds are visualized for all four vGRF characteristics.

Besides the Alcantara et al. (2021)’s and Verheul et al. (2019)’s methods, several other studies have investigated predicting characteristics of the vGRF. One study by Patoz et al. (2023) estimated active peak and contact time from running speed, body mass, stride frequency, and acceleration-based estimates using a machine-learning model. Their best-performing models achieved an RMSE of 0.12 BW and 11.9 ms for active peak and contact time, respectively, which is worse compared to our models. Some other studies have estimated these metrics by approximating the vGRF waveform and calculating the metric from this curve (Donahue and Hahn, 2023; Komaris et al., 2019; Patoz et al., 2022), considered using multiple sensors (Donahue and Hahn, 2023), or the acceleration signal from positions other than the sacrum (Komaris et al., 2019). However, they generally reported higher RMSEs than we achieved. Nevertheless, as every study possesses a unique dataset and often uses a different evaluation metric, it remains hard to directly compare the reported performance without implementing the methods on the same dataset.

The age, running experience, and sports participation varied across the participants. Nevertheless, the older population was underrepresented which might still limit the generalizability of our findings. Furthermore, all running took place on a level treadmill with speeds from 2.22 to 3.89 m/s. As a result, our models might have difficulties in generalizing to different populations, speeds, slopes, or undergrounds. Future studies should investigate the current model’s generalizability to these different conditions. A possible challenge is that vGRF measurements typically require force plates, making it difficult to assess vGRF across varied running surfaces. Moreover, recruiting a diverse participant pool could also be challenging. Investigating the model’s generalizability could be a first step towards adopting the models in the field. A second step requires integrating the models is wearable devices or apps to allow real-time monitoring of the vGRF characteristics in a user-friendly way.

## 5 Conclusion

In this study, we demonstrate that Lasso models can predict vertical ground reaction force (vGRF) characteristics during running from 3D acceleration data collected by a sacral IMU. Our proposed method outperformed the mean-prediction baseline and two established methods for predicting the contact time, active peak, impact peak, and impulse. Overall, our findings indicate the potential utility of this approach as a valuable tool for monitoring select factors related to running-related injuries. Nonetheless, there is a need for further research, mainly toward the accurate prediction of impact peak, and to assess the generalizability to different running conditions.

## Data Availability

The datasets presented in this article are not readily available because of ethical and privacy concerns regarding the participants. Requests to access the datasets should be directed to the corresponding author and the Social and Societal Ethics Committee at KU Leuven.
