# Supplementary material for: Predicting vertical ground reaction force characteristics during running with machine learning
Source: Front Bioeng Biotechnol. 2024 Oct 8;12:1440033. doi: 10.3389/fbioe.2024.1440033 (PMC11493597; doi:10.3389/fbioe.2024.1440033)
Supplement: Supplementary file 1 [file DataSheet1.PDF]

frontiers

# Supplementary Material

## 1 SELECTED TSFRESH FEATURES

The selected tsfresh features are agg_autocorrelation, benford_correlation, change_quantiles, count_above, count_above_mean, count_below, count_below_mean, cwt_coefficients, fft_aggregated, fourier_entropy, friedrich_coefficients, has_duplicate, has_duplicate_max, has_duplicate_min, index_mass_quantile, large_standard_deviation, last_location_of_maximum, last_location_of_minimum, lempel_ziv_complexity, linear_trend, max_langevin_fixed_point, percentage_of_reoccurring_datapoints_to_all_datapoints, percentage_of_reoccurring_values_to_all_values, permutation_entropy, quantile, query_similarity_count, range_count, ratio_beyond_r_sigma, ratio_value_number_to_time_series_length, spkt_welch_density, sum_of_reoccurring_data_points, sum_of_reoccurring_values, sum_values, symmetry_looking, value_count, variance, and variance_larger_than_standard_deviation.

## 2 SUPPLEMENTARY FIGURE

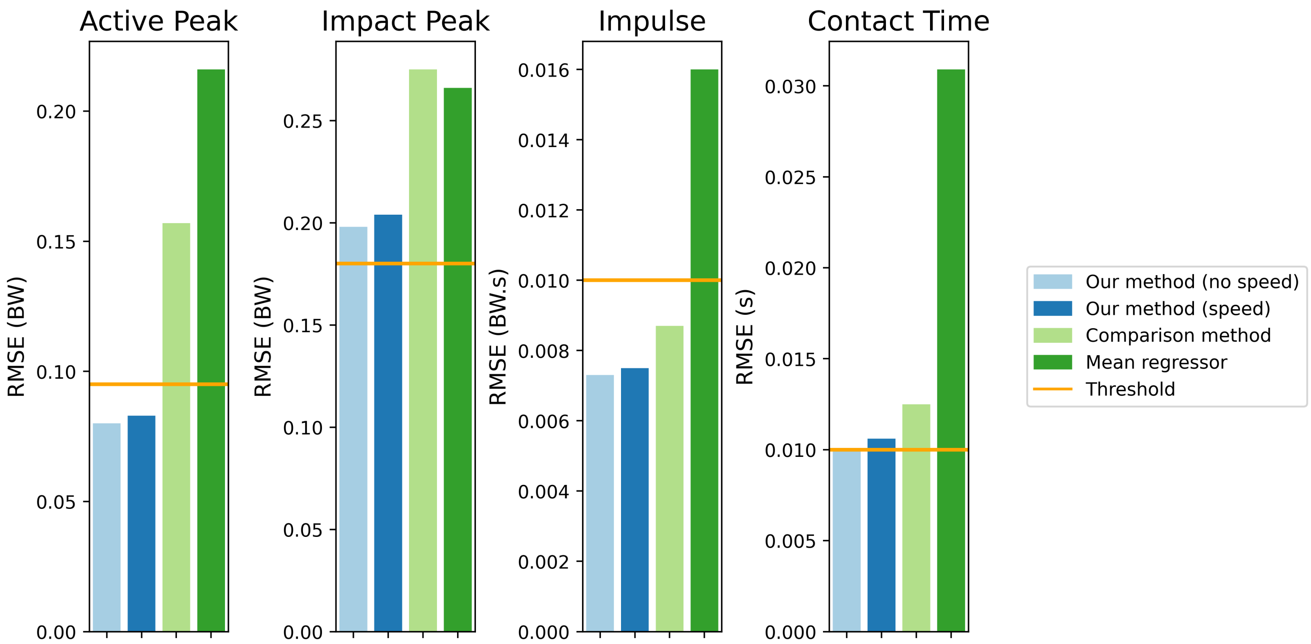


**Figure S1.** Root-mean-square error (RMSE) of our method (with and without speed as input), a comparison method, and a mean regressor to predict active peak, impact peak, impulse, and contact time. The orange line is the threshold determined for a specified application (see text for details).
